# Gene dosage in mammals: characterization of haploid embryonic stem cells

**DOI:** 10.1186/1756-8935-6-S1-O24

**Published:** 2013-03-18

**Authors:** Anton Wutz, Martin Leeb

**Affiliations:** 1 Wellcome Trust - Medical Research Council Stem Cell Institute, Department of Biochemistry, University of Cambridge, Tennis Court Road, Cambridge CB2 1QR, UK

## 

Most animals are diploid but in several cases haploid genomes can support development to some extent. In contrast to fish, haploidy is not compatible with embryonic development in mammals. Albeit, haploid cells have been observed in egg cylinder stage mouse embryos the majority of surviving embryos becomes diploid. Differences between the development of mammals and fish include genomic imprinting and dosage compensation. Both processes are thought to contribute to the loss of haploid embryos.

Haploid embryonic stem cells (ESCs) have recently been derived from mouse parthenogenetic embryos. These cells maintain key properties of mouse ESCs and might overcome existing limitations in developmental genetic approaches in mice. Haploid ESCs have been established through the activation of unfertilized oocytes from a variety of mouse strains and maintain a wide developmental potential in culture and in chimeric mice. Notably, parthenogenetic haploid ESCs possess robust germline competence enabling the production of transgenic mouse strains from genetically modified haploid ESCs. Contribution to the embryo correlates with an efficient gain of a diploid karyotype. We have also observed that differentiation in culture results in diploidization, which likely is the result of endoreduplication and not cell fusion. In contrast to differentiation into embryonic cell types a haploid karyotype is maintained under certain conditions during forced differentiation to extraembryonic cell fates.

Exploring the differentiation potential of a haploid karyotype highlights developmental constraints imposed by evolutionary adaptations specific for the mammalian genome namely genomic imprinting and X chromosome inactivation. Our data would suggest that early embryos possess a remarkable ability to buffer disturbances of both epigenetic processes.

